# *New anti-diabetic drug Morus alba L.* (Sangzhi) alkaloids (SZ-A) improves diabetic nephropathy through ameliorating inflammation and fibrosis in diabetic rats

**DOI:** 10.3389/fmed.2023.1164242

**Published:** 2023-06-09

**Authors:** Caina Li, Quan Liu, Wenming Ji, Yaxin Fu, Hui Cao, Yi Huan, Lei Lei, Xuefeng Gao, Leilei Chen, Cunyu Feng, Lin Zhang, Pingping Li, Yuling Liu, Shuainan Liu, Zhufang Shen

**Affiliations:** ^1^Institute of Materia Medica, Chinese Academy of Medical Sciences and Peking Union Medical College, Beijing, China; ^2^State Key Laboratory of Bioactive Substances and Functions of Natural Medicines, Key Laboratory of Polymorphic Drugs of Beijing, Institute of Materia Medica, Chinese Academy of Medical Sciences and Peking Union Medical College, Beijing, China; ^3^Diabetes Research Center of Chinese Academy of Medical Sciences and Peking Union Medical College, Beijing, China; ^4^Department of Endocrinology, Department of Medical Records, Beijing Tongren Hospital, Capital Medical University, Beijing, China; ^5^Drug Delivery Technology and Novel Formulation, Institute of Materia Medica, Chinese Academy of Medical Sciences and Peking Union Medical College, Beijing, China

**Keywords:** *Morus alba* L. (Sangzhi) alkaloids, diabetic nephropathy, oxidative stress, nitrosative stress, inflammation, renal fibrosis

## Abstract

**Background:**

*Morus alba* L. (Sangzhi) alkaloid (SZ-A) is a new antidiabetic drug approved by the China National Medical Products Administration in 2020. Diabetic nephropathy (DN) is a common diabetic complication and an important cause of morbidity and mortality in patients with diabetes. The effects of SZ-A on DN remain unknown.

**Purpose:**

This study evaluated the effects of SZ-A on DN in Zucker diabetic fatty (ZDF) rats and explored the underlying mechanisms based on nitrosative stress, inflammation, and fibrosis.

**Methods:**

Diabetic ZDF rats were orally administered 100 and 200 mg/kg of SZ-A once daily for 9 weeks. The glucose metabolism and kidney function were assayed. The pathological injury and fibrosis of the kidneys were separately evaluated using hematoxylin and eosin staining and Masson’s staining. The oxidative and nitrosative stress and inflammation were assayed by determining the levels of related indices in the blood and kidneys and quantifying the related gene and protein expression. The expression of transforming growth factor β1 (TGFβ1) gene and protein were assayed by quantitative real-time PCR and immunohistochemistry, respectively. The renal transcriptomics was analyzed using RNA sequencing.

**Results:**

Repeated treatment with SZ-A significantly improved glucose metabolism, dose-dependently decreased the levels of blood urea nitrogen, urinary albumin, and β2-microglobulin, and evidently relieved the renal injury in diabetic ZDF rats. As for the mechanisms, SZ-A remarkably ameliorated systemic nitrosative stress through lowering the levels of blood inducible nitric oxide synthase and nitric oxide, and significantly relieved systemic and renal inflammation by reducing the levels of blood interleukin-1β and monocyte chemoattractant protein-1 (MCP-1) and decreasing the levels of renal C-reactive protein content and expression of *tumor necrosis factor-α* in the kidneys. SZ-A also improved renal fibrosis by lowering the expression of TGFβ1 in the kidneys. Additionally, SZ-A significantly lowered the expression of *stimulator of chondrogenesis 1* in the kidneys.

**Conclusion:**

Repeated treatments with SZ-A significantly ameliorates DN by regulating systemic nitrosative stress, renal inflammation, and renal fibrosis partially through inhibition of the cytokine-NO and TGF-β1 signaling in ZDF rats, providing evidence for the additional application of SZ-A in clinical use for the treatment of DN.

## 1. Introduction

Type 2 diabetes mellitus (T2DM) is a globally common chronic disease that affect numerous people’s lives with a series of complications. Diabetic nephropathy (DN) is a common diabetic complication and an important cause of morbidity and mortality in patients with diabetes by leading to chronic kidney disease, end-stage renal disease, and associated cardiovascular disease. DN is mainly characterized by a spectrum of structural and functional changes in the kidneys, including initial glomerular hyperfiltration, albuminuria, expanded mesangial matrix, interstitial fibrosis, thickened basement membranes, and renal cell damage.

*Morus alba* L. alkaloid (SZ-A) is a new antidiabetic drug approved by the China National Medical Products Administration (Approval number Z20200002). Qu et al. (1) found that SZ-A exhibited hypoglycemic effects similar to acarbose and was accompanied by a lower incidence of treatment-related adverse events and gastrointestinal disorders than acarbose in patients with T2DM. Actually, SZ-A was first discovered as an α-glycosidase inhibitor *in vitro*, and it effectively reduced the peak of postprandial blood glucose following sucrose and starch loading in both healthy and diabetic mice after a single dose (2). Recent studies showed that repeated treatment with SZ-A by gavage significantly improved dyslipidemia, increased the basic and glucose-stimulated secretion of insulin and glucagon-like peptide-1, restored β-cell function, and ameliorated ileal inflammatory injury (3, 4), suggesting that SZ-A possesses extensive pharmacological activities.

Hyperglycemia played a key role in the pathogenesis of DN by inducing the production of reactive oxygen species and reactive nitrogen species that not only directly caused oxidative and nitrosative damage, but also activated a series of inflammatory responses (5). Cao et al. (6) found that SZ-A exerted definitive anti-inflammatory effects by inhibiting the p38 mitogen-activated protein kinase, extracellular regulated protein kinase, and c-Jun N-terminal kinase signaling pathways in bone marrow-derived macrophage and RAW 264.7 cells, further indicating that SZ-A possesses beneficial influences on DN. But the effects and underlying mechanisms remain unknown.

This study aimed to evaluate the effects of SZ-A on DN in Zucker diabetic fatty (ZDF) rats and explore the underlying mechanisms based on oxidative and nitrosative stress, inflammation, and fibrosis.

## 2. Materials and methods

### 2.1. Materials

SZ-A powder (lot number: 201707008) containing a group of effective polyhydroxy alkaloids (50% or more by weight) was provided by the Department of Research and Development of Beijing WeHand-Bio Pharmaceutical Co Ltd. (China). The major alkaloids in SZ-A, including 1,4 -dideoxy-1,4-imino-D-arabinitol, 1-deoxynojirimycin, and fagomine, were analyzed using high performance liquid chromatography-mass spectrometry, and the total polyhydroxy alkaloid content in the SZ-A powder was approximately 63% by weight, mainly including 7% 1,4 -dideoxy-1,4-imino-D-arabinitol, 10.5% fagomine, and 39% 1-deoxynojirimycin (4, 6).

### 2.2. Animal experimental design

Male ZDF-Leprfa/Crl rats (fa/fa) and control rats (fa/+) aged 7–9 weeks were supplied by Beijing Vital River Laboratory Animal Technology Co., Ltd. (Beijing, China) and housed in an environment with a 12 h light/dark cycle with controlled temperature and humidity. All rats were fed with a special diet (Purina, 5,008) and with water *ad libitum*. All the experiments were approved by the Institutional Animal Care and Use Committee of the Institute of Materia Medica (Chinese Academy of Medical Sciences and Peking Union Medical College, Beijing, China) and performed according to the guidelines established by China (GB14925-2001 and MOST 2006a).

The ZDF (fa/fa) rats were divided into the diabetic control group (Con) and SZ-A–treated groups (10 rats per group) in accordance with the non-fasting blood glucose, fasting blood glucose, the increasing ratio of blood glucose at 30 min after oral glucose loading (2 g/kg), blood triglyceride, blood total cholesterol, and body weight. SZ-A was dissolved in water and administrated at the doses of 100 and 200 mg/kg according to our previous study (4). Eight control rats (fa/+) were adopted as a healthy control (Nor). All rats were treated with SZ-A solution or an equivalent volume of water once daily by gavage for 9 weeks. The non-fasting blood glucose (NFBG) and fasting blood glucose (FBG) levels were dynamically monitored. The oral glucose tolerance test (OGTT) was performed after treatment for 5 weeks. The 18 h urine of each rat was collected after treatment for 6 weeks. The fasting blood insulin (FBI) level and glucose stimulated insulin secretion (GSIS) were assayed after treatment for 2 weeks. The glycated hemoglobin (HbA1c) was determined after treatment for 8 weeks. In the end, all rats were sacrificed through cervical dislocation after anesthesia with pentobarbital sodium (60 mg/kg). The whole blood was collected, and the serum was isolated through centrifugation at 6,000 rpm for 10 min; the kidneys were isolated, cut along the longitudinal section, kept at −80°C, and fixed in formalin solution.

### 2.3. Blood glucose and HbA1c measurements

The NFBG and FBG after a 4 h fast with water *ad libitum* were continuously monitored using the glucose oxidase method (Biosino Bio-Technology and Science Inc., Beijing, China). The HbA1c level was measured after treatment for 8 weeks (HOMA Biological Engineering Co. Ltd., Beijing, China).

### 2.4. OGTT, FBI, and GSIS assay

After treatment for 5 weeks, the OGTT was performed. All rats were fasted for 4 h with free access to water, and then the blood glucose was collected before and at 30, 60, and 120 min after an oral glucose loading (2 g/kg body weight). The blood glucose levels were determined and the area under the blood glucose-time curve (AUC) was calculated. After treatment for 2 weeks, the FBI and GSIS were determined. All rats were fasted as above, and then the blood was collected before and at 15 min after an oral glucose loading (2 g/kg body weight). The insulin levels were measured using the mouse ultrasensitive insulin ELISA kit (ALPCO, United States).

### 2.5. Urinary albumin, β2-microglobulin, and blood creatinine and urea nitrogen measurements

After treatment for 6 weeks, the 18 h urine of each rat was collected using a metabolic cage. The levels of albumin and β2-microglobulin in the urine were determined using ELISA kits (β2-microglobulin, Dongge Boye Biotechnology Co. Ltd., Beijing, China; albumin, Abcam, Cambridge, United Kingdom). At the end of experiment, the serum creatinine and urea nitrogen levels were detected using biochemical kits (Nanjing Jiancheng Bioengineering Institute, Nanjing, China).

### 2.6. Histopathological evaluation

The longitudinal section of one kidney from each rat was fixed in formalin solution, embedded in paraffin, and sectioned into a series of 5-μm-thick and 4-μm thick slides. The paraffin sections of 5-μm-thickness were subjected to hematoxylin and eosin (H&E) staining (*n* = 8–10) for the analysis of pathological changes. The proportion of rats with each pathological injury according to the scoring criteria was calculated. Additionally, paraffin sections at 4-μm-thickness were subjected to Masson’s staining for the analysis of renal fibrosis (*n* = 8–10), and the fibrotic tissue area ratio, glomerular fibrosis ratio, and cystic fibrosis ratio were calculated as positive area / tissue area after analyzing 100 renal corpuscles from each slide. The images were captured with OLYMPUS BX43 at 200× magnification. The positive area and tissue area were analyzed with Image-Pro Plus 6.0.

### 2.7. Immunohistochemistry assay

The paraffin sections of 5-μm-thickness were stained with antibodies against transforming growth factor β 1 (TGFβ-1) and collagen I (Servicebio, Wuhan, China). The images were captured and analyzed with Aipathwell software (Servicebio, Wuhan, China). The positive area density and positive area ratio were calculated to assay the expression of TGFβ-1 and collagen I. Positive area density = integrated optical density/tissue pixel area. Positive area ratio = positive area/tissue area.

### 2.8. Oxidative stress, nitrosative stress, and cytokines assays

The levels of inducible nitric oxide synthase (iNOS), nitric oxide (NO), superoxide dismutase (SOD), and total antioxidant capacity (T-AOC) in the serum were determined using the corresponding kits (Nanjing Jiancheng Bioengineering Institute, Nanjing, China). The levels of interleukin-1β (IL-1β) and monocyte chemotactic protein-1 (MCP-1) in the serum were detected by ELISA kits (Abcam, Cambridge, United Kingdom). Approximately 100 mg of kidney tissue was homogenized in cold saline to prepare a 10% homogenate, and the levels of C-reactive protein (CRP), IL-1β, and tumor necrosis factor-α (TNF-α) in the homogenate were assayed using ELISA kits (Abcam, Cambridge, United Kingdom).

### 2.9. Transcriptomic analysis by RNA sequencing

Total RNA of the kidneys was extracted using TRIzol® reagent (Carlsbad, United States). RNA samples of high quality were adopted to construct a sequencing library using the TruSeq™ RNA sample preparation kit (Illumina, San Diego, USA). After being quantified using TBS380, a paired-end RNA-seq sequencing library was sequenced with the Illumina NovaSeq 6,000 sequencer (2 × 150 bp read length). The raw paired-end reads were trimmed and quality-controlled using SeqPrep^1^ and Sickle^2^ to obtain clean reads, which were separately aligned to the reference genome with orientation mode using the HISAT2 software. Next, the mapped reads of each sample were assembled using StringTie (version 2.1.2^3^) in a reference-based approach.

The expression level of each transcript was calculated using the transcripts per million reads method, and then the RSEM software package (version 1.3.3^4^) was used to quantify gene abundances. Differentially expressed genes (DEGs) were analyzed using edgeR (version 3.24.3^5^) with a value of p of <0.05 and |log2FC| of >1. In addition, functional-enrichment analyses of the Kyoto Encyclopedia of Genes and Genomes (KEGG) were performed and analyzed using a Benjamini and Hochberg-corrected value of p of <0.05 compared with the whole-transcriptome background.

### 2.10. Quantitative real-time polymerase chain reaction

Total RNA was extracted from approximately 80 mg of kidney tissue using TRIzol® reagent (Applygen Technologies Inc., Beijing, China), and then subjected to cDNA synthesis using the TransScript® first-strand cDNA Synthesis SuperMix kit (TRANSGEN BIOTECH, Beijing, China). Subsequently, qRT-PCR was performed using the TransStart® Tip Green qPCR SuperMix kit (TRANSGEN BIOTECH, Beijing, China) on a LightCycler® 96 PCR System (Roche, Switzerland) using the following protocol: preincubation at 95°C for 10 min, 55 cycles of amplification at 95°C for 15 s, 60°C for 15 s, and 72°C for 10 s. The DEGs in RNASeq and genes related to oxidative and nitrosative stress, inflammation, and fibrosis in the kidney were assayed. The relative levels of the target mRNAs were calculated using the 2^-ΔΔCt^ method by normalizing to the housekeeping gene β-actin. The primers of the target genes are presented in Table 1.

**Table 1:** The primers of target genes.

mRNAs	Forward primer 5′-3′	Reverse primer 5′-3′
CAT	TCACCTGAAGGACCCTGACA	TCCATCTGGAATCCCTCGGT
GPx1	GCTCACCCGCTCTTTACCTT	TGGAACACCGTCTGGACCTA
SOD1	AGGGCGTCATTCACTTCGAG	CCTCTCTTCATCCGCTGGAC
HO-1	TCTGCAGGGGAGAATCTTGC	TTGGTGAGGGAAATGTGCCA
NOX1	GCTCCAGACCTCCATTTGACA	CTTTCTCAGCGTGTGGTTGC
NOX4	TGTTGGGCCTAGGATTGTGT	CACTGAGAAGTTCAGGGCGT
iNOS	TCCTCAGGCTTGGGTCTTGT	AGAAACTTCCAGGGGCAAGC
TNF-α	TTGAACCAAGCATCACGGGT	TCGCCAGTCCTAACATCAGC
IL-6	AGCGATGATGCACTGTCAGA	GGAACTCCAGAAGACCAGAGC
MCP-1	CAGGTCTCTGTCACGCTTCT	GGCATTAACTGCATCTGGCTG
NF-κB	CCCATACCTTCAAATACTAGAGCA	GTTTGCAAAGCCAACCACCA
TGFβ1	CACTCCCGTGGCTTCTAGTG	GGACTGGCGAGCCTTAGTTT
Collagen I	GGAGAGAGCATGACCGATGG	TTCGATGACTGTCTTGCCCC
Nrg4	CATCCCCAGTGAAGGGCATT	CTTCAGCACTACCCGTTGGT
Tnks	AGGGAGCAAACACCAACGAA	CATAGCTCAGGAGAAGGCGG
Cyp27b1	GGTGAAGAATGGCAGAGGCT	TAGACACAAACACCGAGCCC
Agtr2	AACTGGCACCAATGAGTCCG	GTCAGCCACAGCCAGATTGA
Mlana	TGCCCCGAGAAGAAGTTCAC	CAACCAGGATGCCAATCCCT
Scrg1	TTGCTGTTGGGAACACAAGC	GATCAGCTTCAGGTCGGCTC
Sult1d1	TGGTGAAACAGCACCGTCTT	GGTGCAGTGGATGTTTGCTC
Clec2e	GCAAAATGTCTCAGCGTCGT	AGCAAGGTTCACAGCTCTCC
Hist1h4m	GCATCTCCGGCCTCATCTAC	ACATCCATGGCAGTGACAGT
Gabrr3	GTACATGTGGGTCAGCTCCC	GGAAGTCTGGTCCACGTCAG
Cyp24a1	TCATCTCCCATTCGGCATCG	TCTGGTCCTTGAAGTTCGCC
Akap5	GACCGCAACGATCTGGGTAA	TATCAGCTGTTCACGGCGTT
β-actin	GCAGGAGTACGATGAGTCCG	ACGCAGCTCAGTAACAGTCC

### 2.11. Western blotting analysis

Approximately 80 mg of kidney tissue was lysed in radio-immunoprecipitation assay buffer containing protease inhibitor (Applygen Technologies Inc., Beijing, China) and phosphatase inhibitor (Applygen Technologies Inc., Beijing, China). The protein concentration was determined using a bicinchoninic acid protein assay kit (Applygen Technologies Inc., Beijing, China). Protein (30 ug) from each sample was fractionated using sodium dodecyl sulfate-polyacrylamide gels and transferred onto polyvinylidene difluoride membranes, blocked with 5% non-fat milk for 1 h at room temperature, and incubated with primary antibodies over night at 4°C. The immuno-proteins on the membranes were visualized by enhanced chemiluminescence after incubation with appropriate horseradish peroxidase-conjugated secondary antibodies (ZSGB-BIO, Beijing, China) for 2 h at room temperature. Rabbit anti-phospho-ERK1/2 (Thr202/Tyr204), anti-ERK1/2, and anti-HSP90 were bought from Cell Signaling Technology (United States).

### 2.12. Statistical analysis

All the numerical data are expressed as mean ± SEM and analyzed using one-way ANOVA or non-parametric tests in GraphPad Prism, as well as Excel software. A value of p of <0.05 is representative of statistical significance.

## 3. Results

### 3.1. SZ-A improves glucose metabolism and enhances glucose stimulated insulin secretion in diabetic ZDF rats

**Figure 1:**
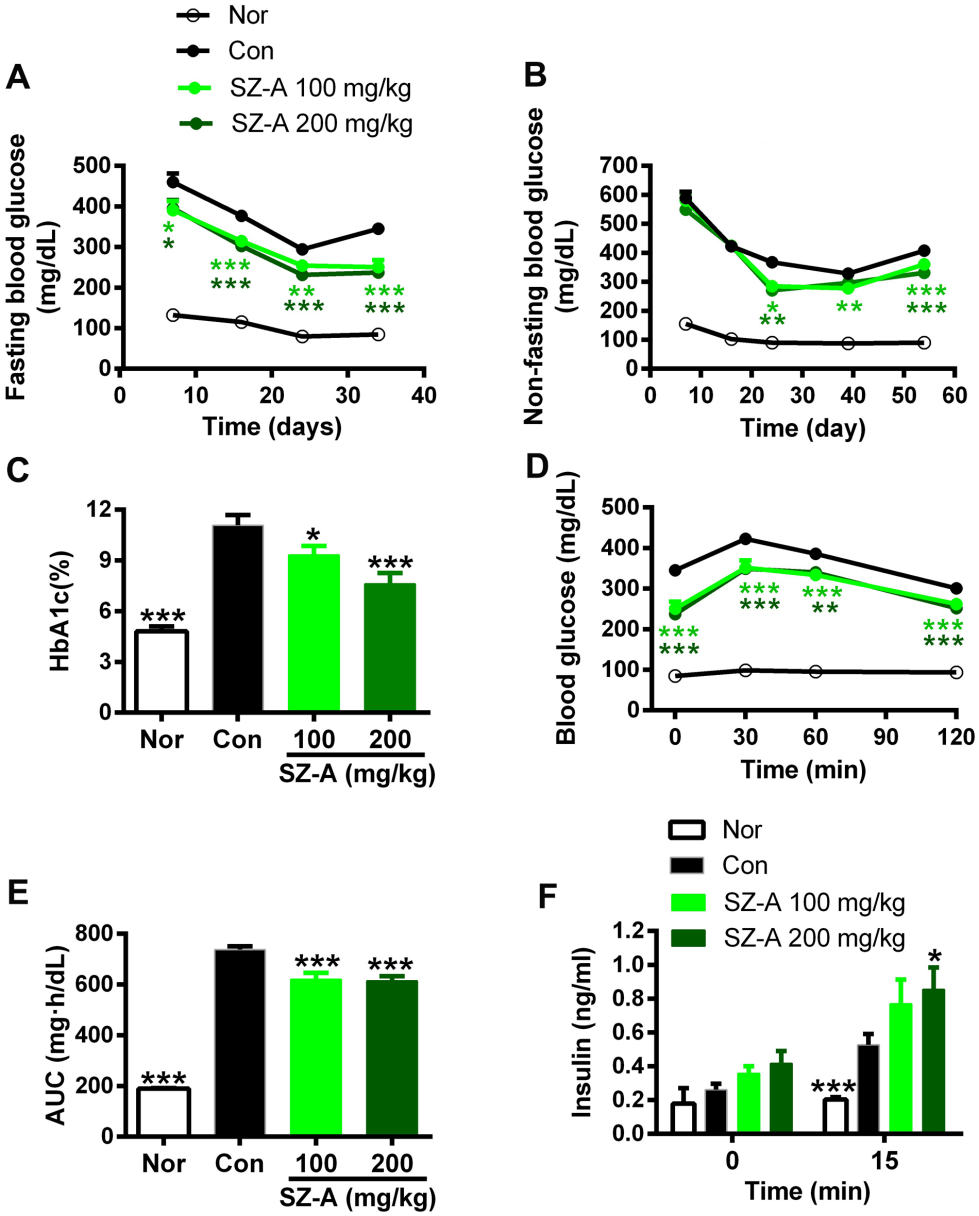
Repeated treatments with SZ-A significantly improved the glucose metabolism in diabetic ZDF rats. **(A,B)** Fasting blood glucose levels and non-fasting blood glucose levels dynamically determined during the treatment. **(C)** The HbA1c level detected after treatment for eight weeks. **(D–E)** Blood glucose levels following oral glucose loading and the area under blood glucose-time curve (AUC) assayed after treatment for five weeks. **(F)** Fasting blood insulin level and blood insulin level at 15 min after an oral glucose loading determined after treatment for two weeks. All data are expressed as mean ± SEM, *n* = 7–10, ***p < 0.001, **p < 0.01, *p < 0.05 vs. Con. Nor, healthy control group; Con, diabetic control group.

In comparison with the Con group, repeated treatments with 100 and 200 mg/kg of SZ-A significantly and continuously decreased the FBG and NFBG levels during the experiment, and dose-dependently reduced the HbA1c level (*p* < 0.05, *p* < 0.001, Figures 1A–C) in ZDF rats. Furthermore, treatment with SZ-A for 5 weeks evidently restrained the blood glucose elevation following oral glucose loading and lowered the area under the blood glucose-time curve (AUC) (Figures 1D,E). Treatment with SZ-A at the dose of 200 mg/kg for 2 weeks significantly increased the glucose-stimulated insulin level in diabetic ZDF rats and had a trend of increasing the fasting blood insulin level (Figure 1F).

### 3.2. SZ-A improves renal function in diabetic ZDF rats

**Figure 2:**
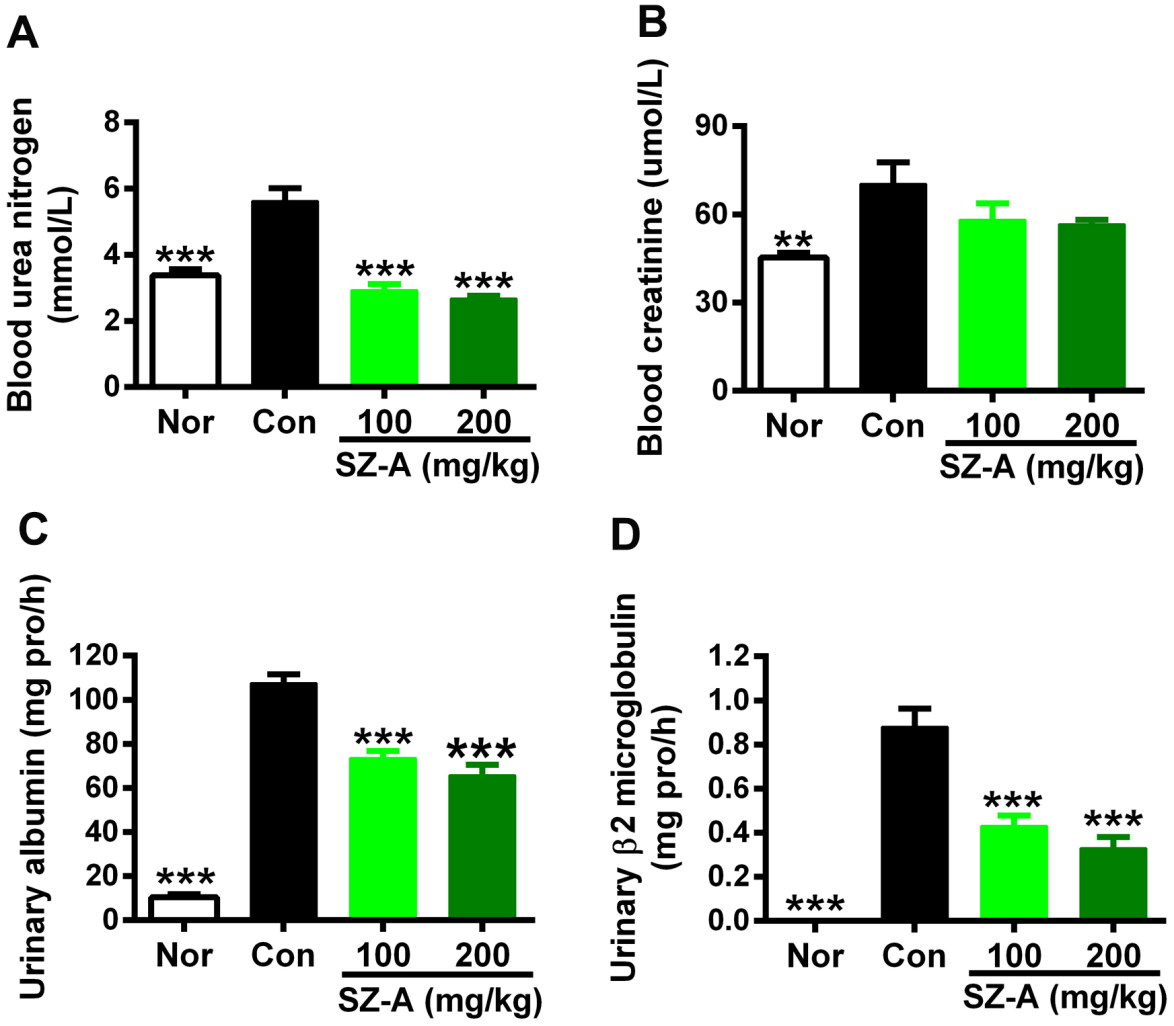
Repeated treatments with SZ-A significantly improved renal function in diabetic ZDF rats. **(A,B)** The levels of blood urea nitrogen and creatinine determined at the end of treatment. **(C,D)** The levels of urinary albumin and β2-microglobulin assayed after treatment for six weeks. All data are expressed as mean ± SEM, *n* = 8–10, ***p < 0.001, **p < 0.01, *p < 0.05 vs. Con. Nor, healthy control group; Con, diabetic control group.

In comparison with the Con group, SZ-A treatment reduced the blood urea nitrogen level in a dose-dependent manner (*p* < 0.001, Figure 2A), and lowered the blood creatinine level by 17.3 and 19.5% at 100 mg/kg and 200 mg/kg doses, respectively (*p* > 0.05, Figure 2B). Furthermore, repeated treatments with SZ-A significantly and dose-dependently decreased the levels of albumin and β2-microglobulin in the urine (*p* < 0.001, Figures 2C,D).

### 3.3. SZ-A ameliorates renal pathological injury and fibrosis in diabetic ZDF rats

**Figure 3:**
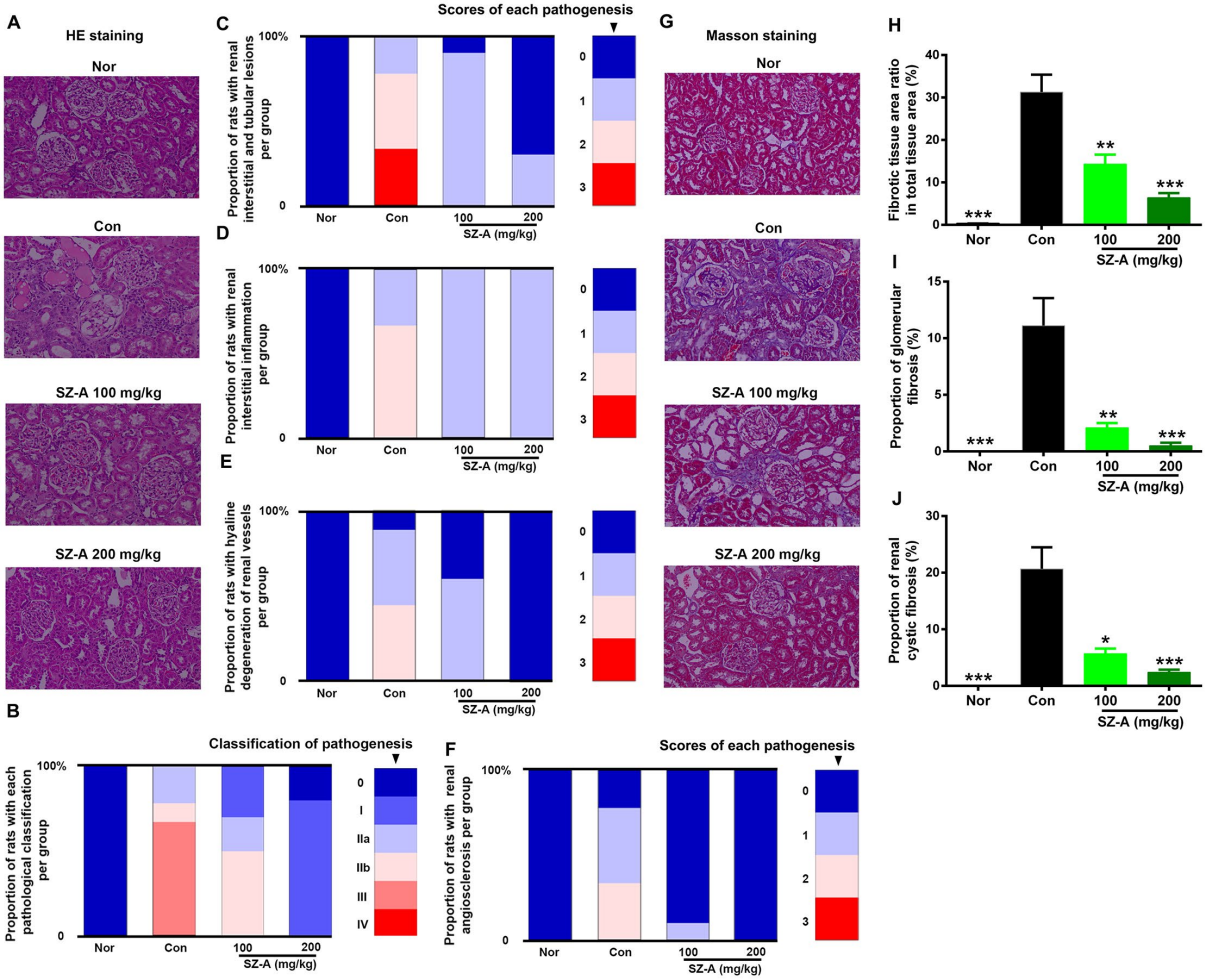
Repeated treatments with SZ-A significantly relieved the renal pathological injuries and fibrosis in diabetic ZDF rats. **(A)** Hematoxylin and eosin (H&E) staining. **(B–F)** Analysis of the proportion of rats with different pathological classifications, renal interstitial and tubular lesions, renal interstitial inflammation, renal vascular hyaline degeneration, and renal angiosclerosis in each group. **(G)** Masson’s staining. **(H–J)** Analysis of fibrotic tissue area ratio in total tissue area, proportion of glomerular fibrosis and proportion of renal cystic fibrosis in each group. All images were taken at 200× magnification. The legends on the right represent the scores of each pathological injury *n* = 8–10. The data for **(H–J)** are expressed as mean ± SEM, ***p < 0.001, **p < 0.01, *p < 0.05 vs. Con. Nor, healthy control group; Con, diabetic control group.

H&E staining was used to evaluate the pathological injury of the kidneys after treatment with SZ-A, and the pathological classification and scoring criteria of each injury are presented in Table 2. Compared with the healthy rats (Nor), the kidneys of the diabetic ZDF rats (Con) suffered significant injuries, including interstitial tubule lesion, interstitial inflammation, vascular hyaline degeneration, and vascular sclerosis, and the related results were displayed as the proportion of rats with each pathological injury per group. Repeated treatments with SZ-A decreased the proportion of rats with serious pathological injuries in a dose-dependent manner in comparison with Con (Figures 3A–F).

**Table 2:** Pathological classification and scoring criteria of H&E staining.

Items	Scores	Scoring criteria
Pathological classification	I	Mild or non-specific thickening of glomerular basement membrane
	IIa	Mild glomerular mesangial hyperplasia
	IIb	Severe glomerular mesangial hyperplasia
	III	Glomerular tuberous sclerosis (at least one definite KW nodule)
	IV	Late diabetic glomerulosclerosis (glomerular sclerosis >50%)
Renal interstitial and tubular lesions	0	No interstitial fibrosis and renal tubular epithelial cell atrophy (IFTA) were found
	1	Interstitial fibrosis and tubular epithelial atrophy (IFTA) were < 25%
	2	Interstitial fibrosis and tubular epithelial atrophy (IFTA) were between 25 and 50%
	3	Interstitial fibrosis and tubular epithelial atrophy (IFTA) were > 50%
Renal interstitial inflammation	0	No
	1	Only inflammatory infiltration associated with IFTA
	2	Inflammatory infiltration except for IFTA
Vascular hyaline degeneration	0	No
	1	Vitreous degeneration of blood vessels at one site
	2	Vitreous degeneration of blood vessels at more than one site
Vascular sclerosis	0	No intimal thickening
	1	Intimal thickening does not exceed the thickness of middle membrane
	2	Intimal thickening exceeds the thickness of middle membrane

Additionally, Masson’s staining was used to evaluate renal fibrosis in ZDF rats following SZ-A treatment (Figure 3G); the renal fibrotic tissue area ratio in total tissue area, proportion of glomerular fibrosis, and proportion of renal cystic fibrosis were calculated. The results show that the diabetic ZDF rats displayed evident renal fibrosis in comparison with Nor. SZ-A treatment significantly and dose-dependently decreased the fibrotic tissue area ratio in total tissue area (Figure 3H), the proportion of glomerular fibrosis (Figure 3I), and the proportion of renal cystic fibrosis (Figure 3J).

### 3.4. SZ-A relieves renal injury by decreasing systemic nitrosative stress in diabetic ZDF rats

**Figure 4:**
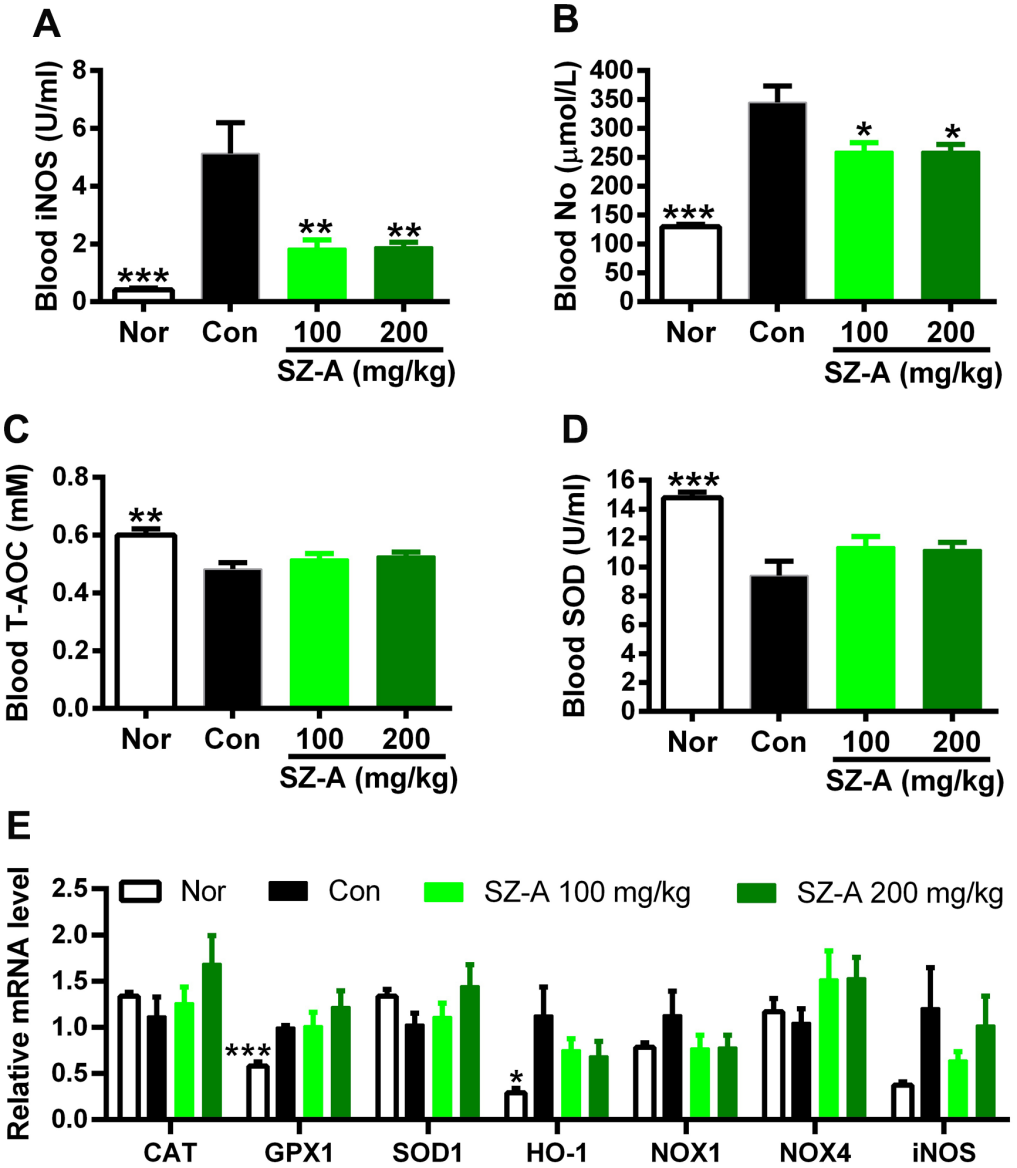
Repeated treatments with SZ-A significantly ameliorated the nitrosative stress in diabetic ZDF rats. **(A)** Blood inducible nitric oxide synthase (iNOS) level. **(B)** Blood nitric oxide (NO) level. **(C)** Blood total antioxidant capacity (T-AOC) level. **(D)** Blood superoxide dismutase (SOD) level. **(E)** The expression of genes associated with oxidative and nitrosative stress. All data are expressed as mean ± SEM, *n* = 3–5 for **(E)** and 7–10 for others, ***p < 0.001, **p < 0.01, *p < 0.05 vs. Con. Nor, healthy control group; Con, diabetic control group; CAT, catalase; GPX1, glutathione peroxidase 1; HO-1, heme oxygenase 1; NOX1, NADPH oxidase 1; NOX4, NADPH oxidase 4.

In comparison with Con, repeated treatments with SZ-A significantly decreased the blood iNOS and NO levels in diabetic ZDF rats (*p* < 0.05, Figures 4A,B), and showed an increasing trend of blood T-AOC and SOD levels (*p* > 0.05, Figures 4C,D). However, SZ-A treatment influenced the expression of genes associated with nitrosative and oxidative stress in the kidneys without significance, such as increasing the expression of catalase (CAT), glutathione peroxidase 1 (GPX1), and SOD1, and decreasing the expression of heme oxygenase 1 (HO-1), NADPH oxidase 1 (NOX1), and iNOS (*p* > 0.05, Figure 4E).

### 3.5. SZ-A ameliorates renal injury by reducing systemic and renal inflammation in diabetic ZDF rats

**Figure 5:**
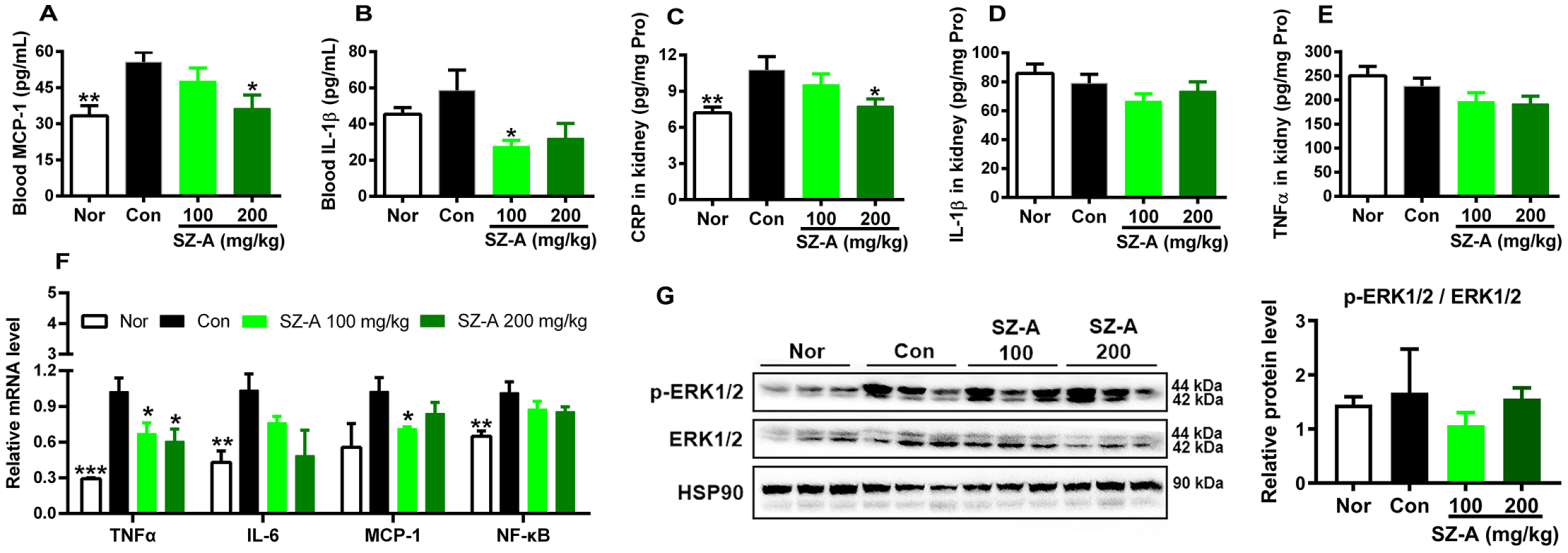
Repeated treatments with SZ-A significantly ameliorated the systemic and renal inflammation in diabetic ZDF rats. **(A,B)** Levels of blood monocyte chemotactic protein-1 (MCP-1) and interleukin-1β (IL-1β) determined with ELISA kits. **(C–E)** Levels of C-reactive protein (CRP), tumor necrosis factor-α (TNF-α), and IL-1β in the kidneys determined with ELISA kits. **(F)** The relative expression of genes associated with inflammation in the kidneys assayed by qRT-PCR. **(G)** The relative expression of ERK1/2 and p-ERK1/2 in the kidneys assayed by western blotting, and HSP90 was detected as internal reference. All data are expressed as mean ± SEM, *n* = 3 for **(G)**, 3–5 for **(F)** and 7–10 for others, ***p < 0.001, **p < 0.01, *p < 0.05 vs. Con. Nor, healthy control group; Con, diabetic control group; IL-6, interleukin-6.

In comparison with Con, repeated treatments with SZ-A significantly reduced the levels of blood MCP-1 and IL-1β and lowered the content of CRP in the kidneys (*p* < 0.05, Figures 5A–C), while it only decreased the contents of IL-1β and TNF-α in the kidneys without significance (*p* > 0.05, Figures 5D,E). In addition, SZ-A treatment also significantly decreased the expression of *TNF-α* in the kidneys (*p* < 0.05), dose-dependently decreased the levels of *interleukin-6 (IL-6)* and *nuclear factor κB* (*NF-κB*) (*p* > 0.05, Figure 5F), and had a trend of lowering the expression of *MCP-1* and phosphorylation of ERK1/2 (*p* > 0.05, Figure 5G).

### 3.6. SZ-A relieves renal fibrosis by decreasing expression TGF-β1 in diabetic ZDF rats

**Figure 6:**
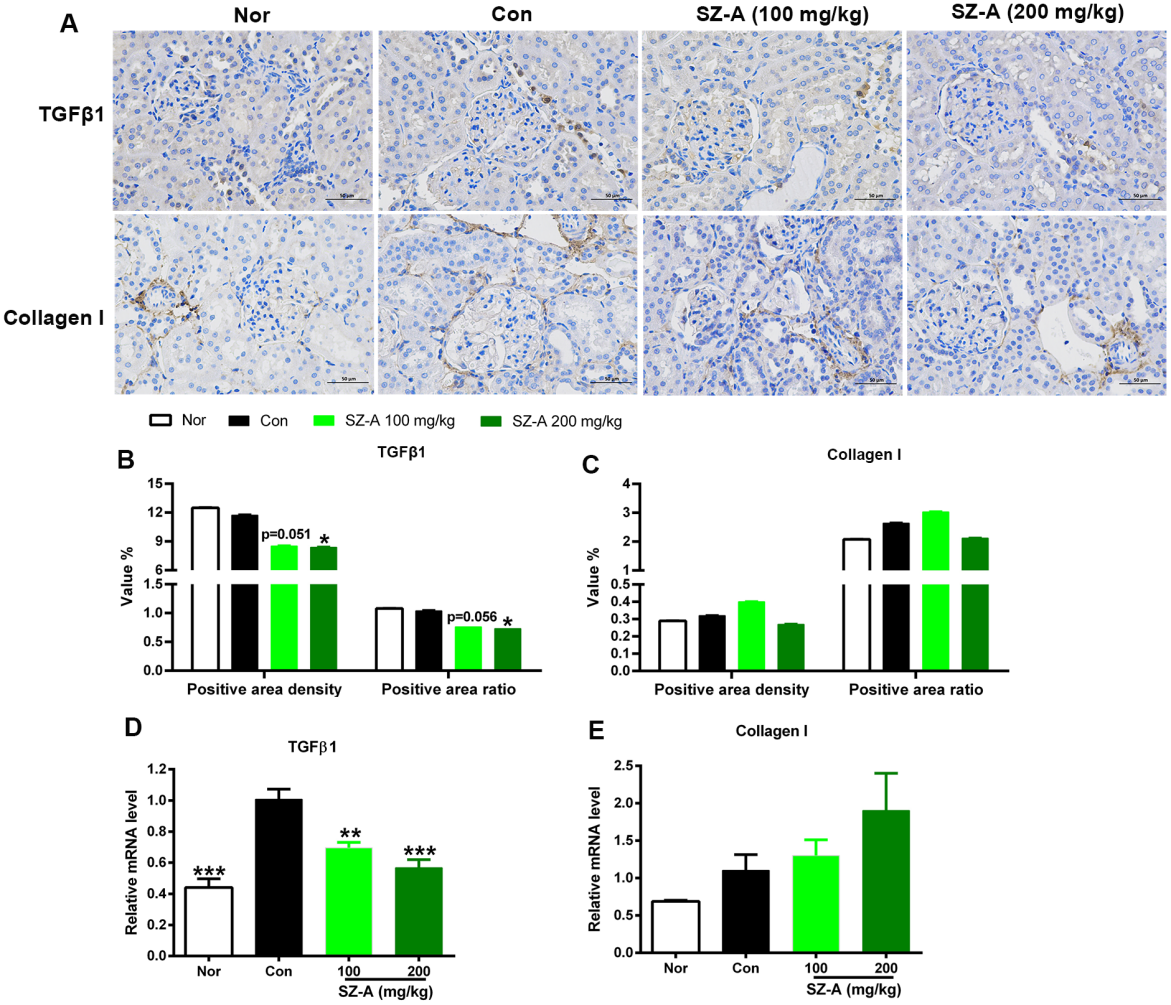
Repeated treatments with SZ-A significantly ameliorated the renal fibrosis in diabetic ZDF rats. **(A)** Immunochemical staining of transforming growth factor β1 (TGF-β1) and collagen I in the kidneys. **(B,C)** Analysis of the positive area density and positive area ratio of TGF-β1 and collagen I. **(D,E)** The expression of TGF-β1 and collagen I in the kidneys assayed by qRT-PCR. All images were taken at 200× magnification. All data are expressed as mean ± SEM, *n* = 4–5 for **(D,E)** and 7–10 for others, ***p < 0.001, **p < 0.01, *p < 0.05 vs. Con. Nor, healthy control group; Con, diabetic control group.

Immunochemical staining was used to evaluate the expression of TGF-β1 and collagen I in the kidneys (Figure 6A), and the positive area density and positive area ratio were calculated. The results show that repeated treatments with SZ-A remarkably decreased the positive area density and positive area ratio of TGF-β1 (Figure 6B), and dose-dependently lowered the expression of *TGF-β1* (*p* < 0.01, Figure 6D). However, SZ-A had no significant effect on the expression of collagen I (Figures 6C,E).

### 3.7. Decreasing expression of Scrg1 may contribute to improving renal inflammation

**Figure 7:**
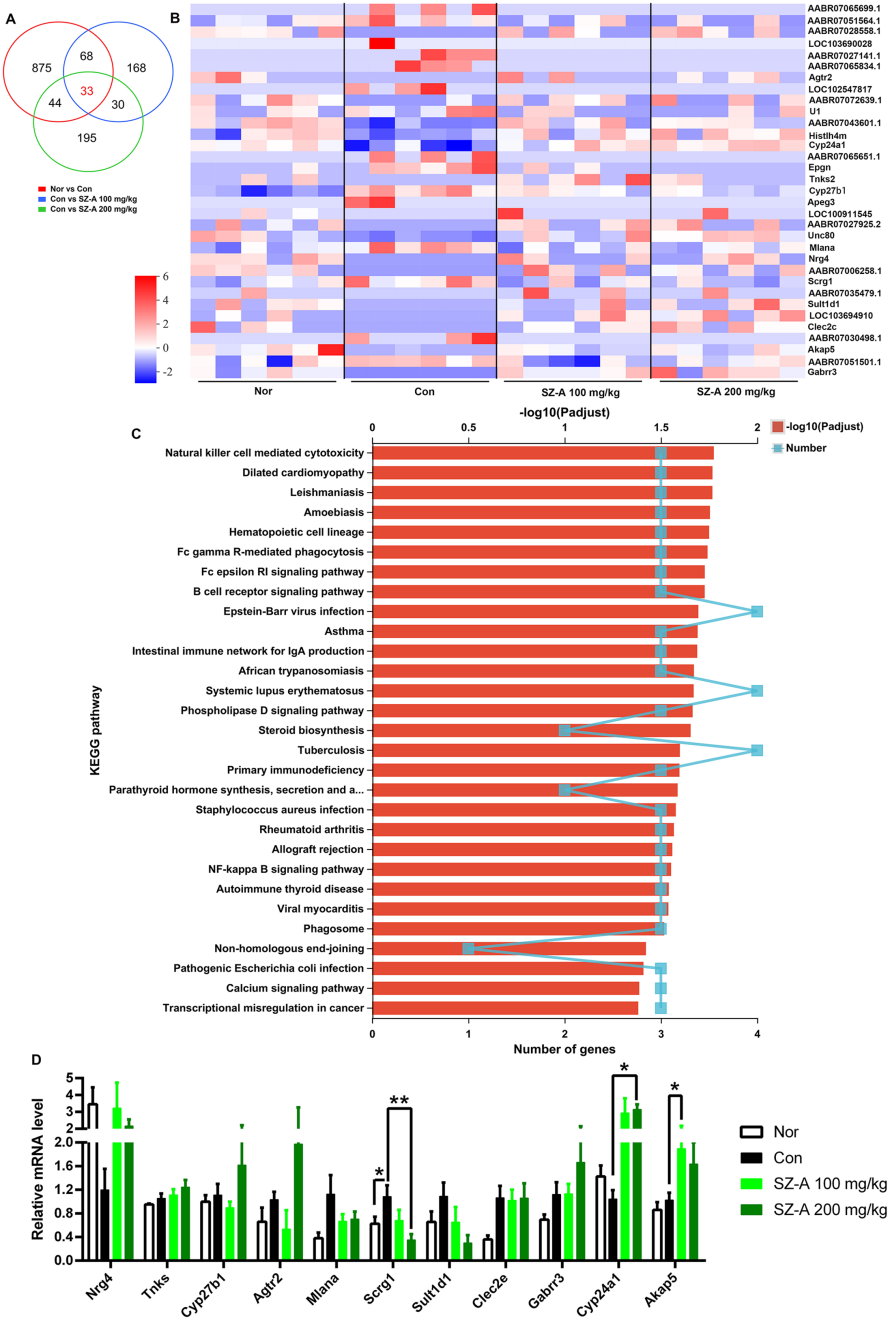
Repeated treatments with SZ-A regulated the renal transcriptomics in diabetic ZDF rats. **(A)** Venn analysis of the DEGs among the comparisons of Nor vs. Con, Con vs. SZ-A (100 mg/kg), and Con vs. SZ-A (200 mg/kg). **(B)** Heatmap of the common 33 DEGs in the Venn analysis. **(C)** Enrichment of the KEGG pathways for the 33 DEGs. **(D)** Relative mRNA levels of some DEGs evaluated using quantitative real-time PCR. Data for **(D)** was expressed as mean ± SEM and *n* = 3–5, *n* = 6 for **(A–C)**. **p < 0.01, *p < 0.05 vs. Con. Nor, healthy control group; Con, diabetic control group; DEGs, differentially expressed genes; KEGG, Kyoto Encyclopedia of Genes and Genomes.

Clean data >6.75 Gb were obtained from each kidney sample in the transcriptomic analysis. Correlation analysis based on gene expression showed that the samples in each group were well-correlated (Supplementary Figure S1). Differential expression analysis revealed 1,020, 299, and 302 DEGs in comparisons of Con vs. Nor, SZ-A 100 mg/kg vs. Con, and SZ-A 200 mg/kg vs. Con, respectively (Supplementary File 1). Venn analysis was performed to search for the common DEGs following treatment with two doses of SZ-A, and 33 common DEGs were detected (Figures 7A,B). Then, the 33 DEGs were subjected to enrichment analysis of the KEGG pathway; 54 KEGG pathways were enriched and 29 of the KEGG pathways reached significance (corrected value of p of <0.05), including immune signaling pathways, such as natural killer cell-mediated cytotoxicity, Fc gamma R-mediated phagocytosis, and the Fc epsilon RI signaling pathway, as well as the inflammatory NF-κB signaling pathway (Figure 7C). Some of the DEGs were verified using qRT-PCR, and the results show that SZ-A treatment significantly lowered the expression of *stimulator of chondrogenesis 1* (*scrg1*) (*p* < 0.05) and increased the expression of cytochrome P450 enzyme *cyp24a1* and *A-kinase anchoring protein 5* (*akap5*) in the kidneys (p < 0.05) (Figure 7D), which were consistent with the results of RNASeq.

## 4. Discussion

Studies have shown that hyperglycemia is the “culprit” of DN, and seriously controlling blood glucose fluctuation has proven effective in delaying the occurrence and progression of DN. SZ-A is a new antidiabetic drug that is extracted from the mulberry branch and mainly contains a series of polyhydroxy alkaloids and glycosides (≥ 50% by weight) (7). Yang et al. (8) reported that the kidney was one of the major distribution tissues of 1,4 -dideoxy-1,4-imino-D-arabinitol, fagomine, and 1-deoxynojirimycin, which are three important polyhydroxy alkaloids in SZ-A, after oral administration of 40 mg/kg dose in rats, pointing out that SZ-A may possess renal benefits.

Generally, microalbuminuria is an indicator of DN, and predicts cardiovascular mortality in patients with diabetes (9). The levels of blood creatinine and urea nitrogen are also common indicators for evaluating renal function. Repeated administration of SZ-A not only dose-dependently constrained the blood glucose variation in ZDF rats, but also significantly lowered the levels of blood urea nitrogen and urinary albumin and reduced the blood creatinine level by 19.5% at the dose of 200 mg/kg. Furthermore, both H&E and Masson’s staining showed remarkable improvement of renal pathogenesis after treatment with SZ-A. Therefore, the study indicates that SZ-A may ameliorate or delay the development and progression of DN in the clinic and confer certain benefits on the kidneys when exerting antidiabetic effects.

To date, many promising therapeutic targets of DN have been revealed by identifying the pathological mechanisms of oxidative stress, inflammation, and fibrosis in preclinical studies (10). Improving epithelial-mesenchymal transition by restraining the NADPH oxidase/ROS/ERK1/2 pathway were reported to be associated with the renal protection of mulberry (11). In the present study, SZ-A treatment showed no significant effects on the oxidative stress-related enzymes, such as T-AOC, SOD1, CAT, GPX1, NOX1, or NOX4, but significantly decreased the blood iNOS and NO levels, although it only showed a decreasing trend of renal *iNOS* expression.

As a short-lived gaseous lipophilic molecule, NO is produced in almost all tissues under the action of nitric oxide synthase (NOS). Studies showed that NO plays various physiological roles in the kidneys by interfering with multiple and physiologically critical steps of nephron function (12). Sugimoto et al. (13) found that induction of iNOS and production of NO accompanied with augmented expression of TNFα and advanced glycation end products (AGEs) in streptozotocin-induced diabetic rat glomeruli and proposed that AGE-cytokine-NO sequence pathway may be a major mechanism of DN. Although there were confusing and contradictory reports about the role of NO in renal injury under different study conditions, it is reasonable to conclude that early nephropathy in diabetes is associated with increased intrarenal NO production (14). Therefore, this study indicated that SZ-A can improve renal function by relieving NO production in diabetic ZDF rats.

Inflammation has been shown to be a cardinal pathogenetic mechanism in DN. Proinflammatory cytokines are produced in multiple renal cells, including glomerular, endothelial, tubular, and mesangial cells. IL-1, IL-6, MCP-1, and TNFα are considered to be the main regulators of inflammation and have central roles in the inflammatory cascade leading to DN (15, 16). NF-κB is a ubiquitous transcription factor that is activated by inflammatory cytokines, which in turn participate in regulating cytokines. In addition, CRP is an essential inflammatory marker and mediator in chronic diseases such as DN. Lai et al. (17) found that CRP is also pathogenic as it triggers renal inflammation and fibrosis, and activation of NF-κB and TGF-β signaling was involved in the process (18). In the present study, SZ-A significantly decreased the levels of systemic and renal inflammatory factors, including IL-1β, MCP-1, CRP, and TNFα. Thus, this study indicates that SZ-A relieves renal inflammation mainly through cytokine-NO pathway in diabetic ZDF rats.

Interstitial fibrosis is a crucial stage and eventually culminates in renal failure in the progression of DN, which is characterized by excessive deposition/turnover of extracellular matrix (ECM) components such as collagen type I and III (19). TGF-β is demonstrated to be the key driver of renal fibrosis, especially TGF-β1, which can induce renal fibrosis *via* activation of Smad-based and non-Smad-based signaling pathways, leading to excessive production of ECM and inhibition of ECM degradation (20). Repeated treatment with SZ-A dose-dependently inhibited the expression of TGF-β1 in the kidneys of diabetic ZDF rats, indicating that the primary mechanism of SZ-A improving renal fibrosis lies in inhibiting TGF-β1 signaling.

Additionally, RNA-Seq was performed to explore the extensive mechanisms, and three DEGs, scrg1, akap5, and cyp24a1, were verified to be significantly influenced by the treatment with SZ-A. Scrg1 is a small protein rich in cysteine that is reported to be involved in the host response to stress and immune regulatory pathways (21). Liu et al. (22) reported that *scrg1* was upregulated in osteoarthritis synovitis and was correlated with increased immune response; thus, scrg1 has been identified as a potential therapeutic target for human synovial inflammation. AKAP5 belongs to a group of regulatory proteins that can scaffold various kinases and phosphatases in excitable cells (23). There are reports that AKAP5 modulated glucose homeostasis by modulating Ca^+^ currents and oscillatory production of cAMP to organize insulin secretion (24), but also mobilized cPKC-dependent cardiac glucotoxicity (25). There have been no reports about the role of akap5 in renal function. In the present study, SZ-A significantly decreased the expression of *scrg1* and increased the expression of *akap5* in the kidneys of diabetic ZDF rats, suggesting a possible role for them in regulating renal function in diabetic ZDF rats. CYP24A1 functions to maintain physiological levels of 1, 25-dihydroxyvitamin D_3_ in the kidneys. One study found that the upregulation of CYP24A1 expression decreased vitamin D, which is related to chronic kidney disease; therefore, blocking CYP24A1 activity might be an approach for correcting vitamin D deficiency in patients with chronic kidney disease (26). However, SZ-A significantly increased the expression of *cyp24a1* in the kidneys of diabetic ZDF rats. Thus, the role of cyp24a1 still needs further research.

## 5. Conclusion

In summary, repeated treatments with SZ-A significantly improved renal function through ameliorating nitrosative stress, inflammation, and fibrosis in diabetic ZDF rats, and the underlying mechanisms involve inhibition of the cytokine-NO and TGF-β1 signaling pathways. Decreasing renal *scrg1* may also participate in the benefits of SZ-A, but further validation is needed. This study suggests that SZ-A may provide a new alternative for the treatment of DN in the future.

## Data availability statement

The sequencing data can be found in online repository NCBI SRA (PRJNA933968).

## Ethics statement

The animal study was reviewed and approved by the Institutional Animal Care and Use Committee of the Institute of Materia Medica (Chinese Academy of Medical Sciences and Peking Union Medical College, Beijing, China).

## Author contributions

CL: conceptualization, investigation, methodology, data curation, formal analysis, and writing – original draft. QL: investigation, methodology, and data curation. WJ: investigation and formal analysis. YF: investigation and data curation. HC and YH: data curation and funding acquisition. LL: data curation. XG, LC, and CF: investigation. LZ and PL: funding acquisition. YL: supervision and writing – review and editing. SL: conceptualization, supervision, writing – review and editing, and funding acquisition. ZS: supervision and writing – review and editing. All authors contributed to the article and approved the submitted version.

## Funding

The work was supported by the Natural Science Foundation of Beijing Municipality (no. 7202137), the CAMS Initiative for Innovative Medicine (CAMS-I2M) (nos. 2021-I2M-1-026, 2022-I2M-JB0011 and 2022-I2M-2-002), the National Natural Science Foundation of China (nos. 81973379 and 81900480), the Beijing Outstanding Young Scientist Program (no. BJJWZYJH01201910023028), and the Fund for Beijing Science and Technology Development of TCM (No. JJ-2020-25).

## Conflict of interest

The authors declare that the research was conducted in the absence of any commercial or financial relationships that could be construed as a potential conflict of interest.

The handling editor QL declared a shared parent affiliation with the author LZ at the time of review.

## Publisher’s note

All claims expressed in this article are solely those of the authors and do not necessarily represent those of their affiliated organizations, or those of the publisher, the editors and the reviewers. Any product that may be evaluated in this article, or claim that may be made by its manufacturer, is not guaranteed or endorsed by the publisher.

## Footnotes

^1^ https://github.com/jstjohn/SeqPrep

^2^ https://github.com/najoshi/sickle

^3^ https://ccb.jhu.edu/software/stringtie/

^4^ http://deweylab.biostat.wisc.edu/rsem/

^5^ http://bioconductor.org/packages/stats/bioc/edgeR/
