# Supplementary figures and images for: New anti-diabetic drug Morus alba L. (Sangzhi) alkaloids (SZ-A) improves diabetic nephropathy through ameliorating inflammation and fibrosis in diabetic rats
Source: Front Med (Lausanne). 2023 Jun 9;10:1164242. doi: 10.3389/fmed.2023.1164242 (PMC10289017; doi:10.3389/fmed.2023.1164242)

## Correlation between samples

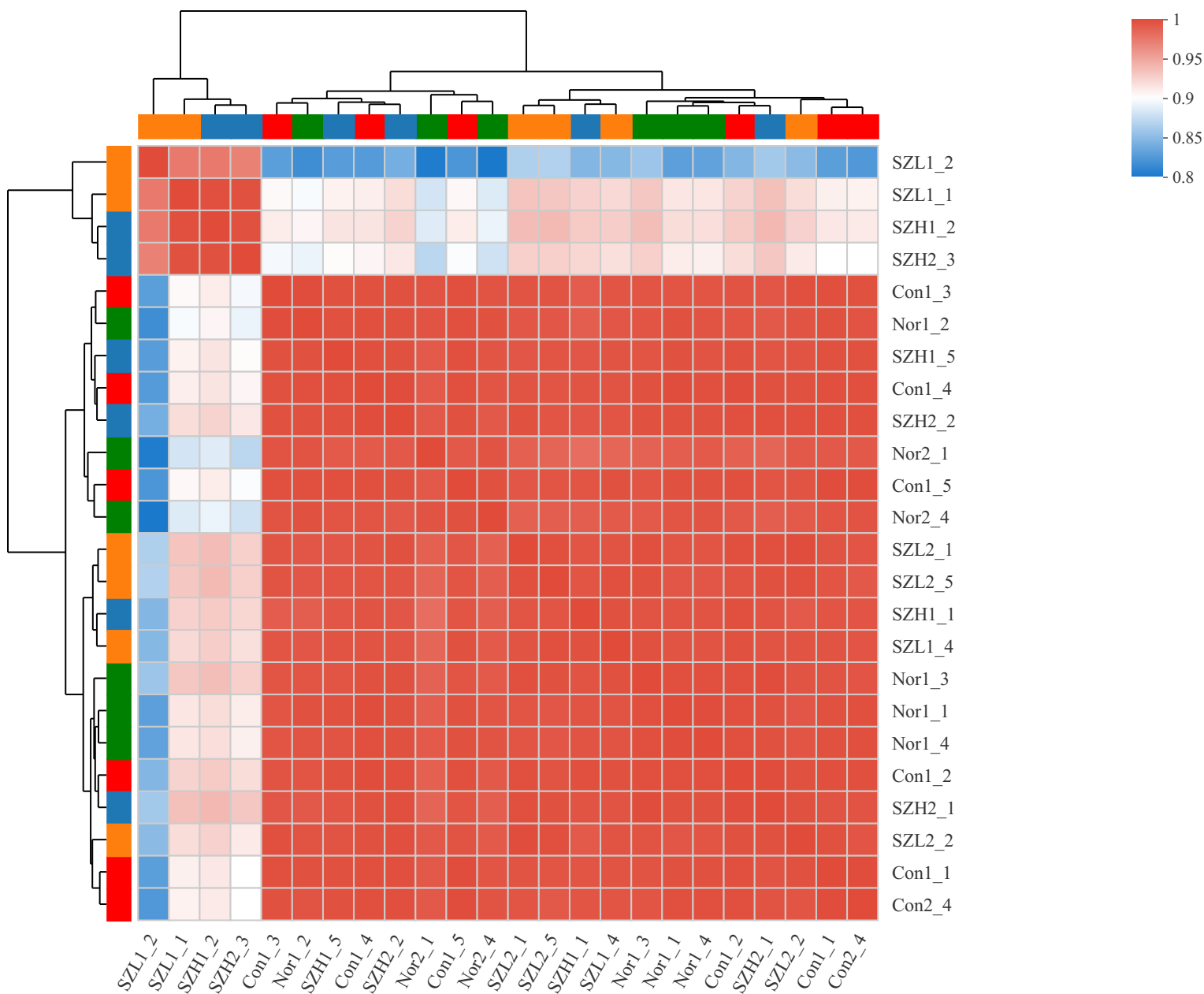

Supplement: Supplementary file 1 [file Data_Sheet_1.PDF]
